# Comparative Effects of Compound Enzyme and Antibiotics on Growth Performance, Nutrient Digestibility, Blood Biochemical Index, and Intestinal Health in Weaned Pigs

**DOI:** 10.3389/fmicb.2021.768767

**Published:** 2021-10-29

**Authors:** Zhiqing Li, Lizi Tang, Nian Liu, Fan Zhang, Xiang Liu, Qian Jiang, Jiashun Chen, Xiaokang Ma

**Affiliations:** College of Animal Science and Technology, Hunan Agricultural University, Changsha, China

**Keywords:** compound enzymes, growth performance, nutrient digestibility, serum biochemical profiles, intestinal health, weaned piglets

## Abstract

This experiment aims to explore the effects of compound enzyme preparation substituting chlortetracycline on growth performance, serum immune markers, and antioxidant capacity and intestinal health in weaned piglets. A total of twenty-four 28-day-old “Duroc × Landrace × Yorkshire” weaned piglets with an average initial weight of 7.25 ± 0.25 kg were randomly divided into three groups according to their body weight, with eight replicates in each group and one pig in each replicate. The three dietary treatments were basal diet (CON), basal diet + 1,000 mg/kg compound enzyme preparation (cellulase 4,000 IU/g, α-amylase 1,000 IU/g, β-glucanase 150 IU/g, and neutral protease 3,000 IU/g, CE), and basal diet + 75 mg/kg chlortetracycline (CTC). The animal experiment lasted for 28 days and was divided into two stages: the early stage (0–14 days) and the late stage (15–28 days). The results showed that (1) compared with the CON, the CE and CTC significantly increased the ADG of weaned piglets during the early and whole period of experiment (*p* < 0.05), decreased the F:G in the whole experiment period (*p* < 0.05), and diarrhea rate in the early stage (*p* < 0.01). (2) Compared with the CON, the apparent total tract digestibility of ADF and NDF was significantly increased in pigs fed the CE diet in the early and late stages of experiment (*p* < 0.05) with no significant difference compared with the CTC. (3) Compared with the CON, the concentrations of serum IgA and SOD in weaned piglets were significantly increased in the CE group in the early stage of the experiment (*p* < 0.05). (4) Compared with the CON group, the acetic acid, propionic acid, and total VFA contents in cecum and colon segments were elevated in the CE group (*p* < 0.05) with no significant difference compared with the CTC. (5) Compared with the CON group, the villus height of duodenum and jejunum and the ratio of villus height to recess depth of ileum were increased in the CE and CTC group (*p* < 0.05). (6) Compared with the CON group, the abundance of *Lactobacillus* significantly increased (*p* < 0.01) while the abundance of *Escherichia coli* decreased in the CE group and CTC group (*p* < 0.01). In conclusion, CE preparation instead of CTC can significantly improve the nutrient digestibility, the immunity, antioxidant capacity, and intestinal health of pigs, which may contribute to the improved growth performance of piglets.

## Introduction

In the pig industry, weaning stress of piglets has always been a huge problem. Piglets have a series of problems such as diarrhea, anorexia, and decreased growth performance due to immature digestive tract, low immune function, and dietary changes (Li et al., 2021). In the past, antibiotics were commonly used in piglets to reduce weaning stress, but bacterial resistance to antibiotics has become a global threat to animals and public health (Teale and Borriello, 2021). The inclusion of antibiotics in animal diets is a controversial issue worldwide (Zainab et al., 2020). China stopped using antibiotics as feed additives in July 2020. Therefore, it is necessary to develop new feed additives to replace antibiotics and improve the health condition of pigs and its product quality.

Since antibiotics were banned, enzymes used in pig diets have been widely accepted (Willamil et al., 2012). It has been reported that dietary supplementation of compound enzyme containing amylase, protease, and xylanase can promote the growth of piglets by improving nutrient digestibility and regulating intestinal flora (Yi et al., 2013). Since cereal feeds contain large amounts of soluble non-starch polysaccharides (NSP), it not only reduced nutrient digestibility but also induced inflammation in pigs (Woyengo et al., 2009; Vila et al., 2018). Moreover, starch particles embedded in cereal protein matrix are not easily decomposed by starch degrading enzymes (Zaefarian et al., 2015). The adverse effects of NSP and cellulose could be alleviated by adding cellulase, α-amylase, and β-glucanase to cereal grain-based diets. In addition, supplementation of cereal grain-based diets with protease for pigs may increase the degradation of protein that interacts with NSP and starch, thereby increasing nutrient digestibility.

At present, most studies mainly explore the effects of different enzyme preparations on barley, oats, or Distillers Dried Grains with Solubles (DDGS) as the basic diet, while there are few studies investigating whether enzyme preparations can improve the utilization efficiency of corn–soybean meal diet. Therefore, this experiment was conducted to evaluate the effects of adding a compound enzyme preparation composed of NSP enzyme, amylase and protease to replace antibiotics in corn-soybean meal diet on growth performance, serum immune markers, and antioxidant capacity and intestinal health of weaned piglets. It provides scientific basis and theoretical basis for the application of compound enzyme preparation to replace antibiotics in animal production.

## Materials and Methods

The compound enzyme used in this study contained amylase, protease, and xylanase. The compound enzyme preparation is provided by DuPont, Inc. The main components are cellulase (4,000 IU/g), α-amylase (1,000 IU/g), β-glucanase (150 IU/g), and neutral protease (3,000 IU/g). According to the results of *in vitro* test (Long et al., 2021) and the company‘s recommendation, the supplemental amount was 1,000 mg/kg.

### Animal Treatment and Experimental Design

Twenty-four 28-day-old weaned piglets (Duroc × Landrace × Large White) with an average initial body weight of 7.25 kg were randomly allocated to three groups with eight replicates per group and one piglet per replicate according to gender and body weight in a randomized complete block design. The three experimental diets were a corn–soybean meal basal diet (CON), a basal diet supplemented with 1,000 mg/kg compound enzyme preparation (CE) and a basal diet supplemented with 75 mg/kg chlorotetracycline preparation (CTC). The animal experiment lasted for 28 days, which was divided into early (0–14 days) and late (15–28 days) stages. The nutrient composition of diets (Table 1) met or exceeded the requirements of NRC (2012).

**TABLE 1:** Ingredient composition and nutrient levels of the experimental diets (%, as-fed basis).

Items	Content (%)
**Ingredients**	
Corn	54.75
Soybean meal	19.00
Full-fat soybean powder	10.00
Fish meal	5.00
Whey powder	6.15
Soybean oil	1.50
Dicalcium phosphate	0.90
L-Lysine-HCl	0.48
L-Threonine	0.05
DL-Methionine	0.10
L-Tryptophan	0.02
Salt	0.30
Limestone	0.50
Premix^a^	1.00
Cr_2_O_3_	0.25
Total	100.00
**Calculated nutrients**	
Digestible energy (MJ/kg)	14.64
Crude protein	20.15
Lysine	1.38
Methionine	0.82
Methionine + cysteine	1.01
Threonine	0.97
Tryptophan	0.25
Calcium	0.80
Total phosphorus	0.73

*^a^The premix provided the following (per kilogram of complex feed): Vitamin A, 12,000 IU; Vitamin D, 2,500 IU; Vitamin E, 30 IU; Vitamin B12, 12 μg; Vitamin K, 3 mg; d-pantothenic acid, 15 mg; nicotinic acid, 40 mg; choline chloride, 400 mg; Mn, 40 mg; Zn, 100 mg; Fe, 90 mg; Cu, 8.8 mg; I, 0.35 mg; Se, 0.3 mg.*

These experiments were conducted in accordance with Chinese guidelines for animal welfare and experimental protocols, and all animal procedures were approved by the Committee of Animal Care at Hunan Agricultural University (Changsha, China) (Permit Number: CACAHU 2021-01106). The animal experiments were carried out in the Animal Test Base of Hunan Agricultural University. All the piglets were housed on a net bed with a leaky floor and had free access to feed and water. The piglets were dewormed and immunized according to routine procedures and pig houses were cleaned and disinfected regularly. The ambient temperature is automatically adjusted by the thermostatic controller and windows are regularly opened for ventilation.

## Detection Indicators

### Growth Performance and Diarrhea Rate

On days 0, 14, and 28, the body weight and feed intake of pigs were recorded to calculate average daily gain (ADG), average daily feed intake (ADFI), and feed to weight gain ratio (F:G). From days 0 to 28, the health status and mortality of each piglet were recorded. The occurrence of diarrhea and fecal score of each pig was visually assessed and evaluated twice a day by trained observers blinded to the treatments according to the method of Long et al. (2017). In brief, the severity of diarrhea was assessed using a scoring system as shown below: 1 = hard stool; 2 = slightly soft feces; 3 = partially formed soft feces; 4 = loose semi-liquid feces; 5 = watery mucous feces. Pigs are identified as having diarrhea when the average score exceeds 3 points. Diarrhea rate was determined mainly based on the average score of feces and the formula was: diarrhea rate (%) = days of diarrhea number of pigs with diarrhea/(test days × total number of pigs).

### Nutrient Digestibility

During this experiment, a total of 1 kg of feed samples were collected weekly. The dry matter (DM), crude protein (CP), acid detergent fiber (ADF), and neutral detergent fiber (NDF) in feed and fecal samples were measured according to AOAC (2012). The apparent total tract digestibility (ATTD) was calculated as follows:


$$\begin{array}{r} \mathrm{ATTD}\;\mathrm{nutrient}=1-(\mathrm{Cr}{}_{\mathrm{diet}}\cdot \mathrm{Nutrient}{}_{\mathrm{feces}})/\\ (\mathrm{Cr}{}_{\mathrm{feces}}\cdot \mathrm{Nutrient}{}_{\mathrm{diet}}). \end{array}$$

### Serum Assays

On days 14 and 28, blood samples were collected by anterior vena cava puncture in each treatment, centrifuged at 3,000 *g* at 4°C for 15 min to obtain the serum, and stored at –20°C until analysis. Serum immunoglobulin concentration was determined by using an ELISA kit (A) following the manufacturer’s instructions (Cusabio Biotechnology Co., Ltd., Wuhan, China). Moreover, the contents of total antioxidant capacity (T-AOC), superoxide dismutase (SOD), glutathione peroxidase (GSH-Px), and malondialdehyde (MDA) in serum were determined using a spectrophotometer (Leng Guang SFZ1606017568, Shanghai, China) following the manufacturer’s instructions (Nanjing Jiancheng Bioengineering Institute, Nanjing, China).

### Volatile Fatty Acids Analysis

Digesta samples from cecal, colonic, and ileum segments were collected on day 28 to determine volatile fatty acid (VFA) content (*n* = 8). All samples were frozen in a –80°C freezer immediately after collection. VFA content of digesta was determined using a HP 5, 890 gas chromatograph (HP, Pennsylvania, United States) according to the method of Long et al. (2017).

### Morphology of Small Intestine

At the end of the animal experiment, 24 pigs (8 pigs per treatment) were slaughtered and the proximal, middle, and distal part of the small intestine from the gastric pylorus to the ileo-cecal valve were obtained to analyze the morphological changes of duodenum, jejunum, and ileum. The samples of small intestine were fixed in neutral-buffered formalin and processed by the standard paraffin method. Small intestine sections (9–10 cm) were stained with hematoxylin and eosin. Measurements of villus height and crypt depth were taken only from sections where the plane of section ran vertically from the tip of villus to the base of an adjacent crypt by using a light microscope. Ten well-oriented villus × 3 sections of each pig were used to determine these indices (Wang et al., 2021).

### Microbiota Analysis by 16S RNA

Total genome DNA was extracted from cecal digesta samples using the QIAamp Fast DNA Stool mini kit (Qiagen, Hilden, Germany) and checked with 1% agarose gel. The DNA concentration and purity were determined with Nano Drop 2,000 UV-vis spectrophotometer (Thermo Fisher Scientific, Wilmington, United States). The specific primer with the barcode (16S V3-V4) were amplified by an ABI Gene Amp^®^ 9,700 PCR thermocycler (ABI, CA, United States). Then, the PCR products were extracted, purified, and quantified.

Paired-end sequencing was performed on an Illumina MiSeq PE300 platform/NovaSeq PE250 platform (Illumina, San Diego, United States). The raw 16S rRNA gene sequencing reads were demultiplexed, quality-filtered and merged according to previous studies (Magoč and Salzberg, 2011; Chen et al., 2018). The complexity of species diversity was evaluated with ACE and Chao richness estimators and diversity indices of Shannon and Simpson (Edgar, 2013). β-diversity was evaluated using principal component analysis (PCA) based on the Euclid distance. The significant differences between samples were evaluated by analysis of similarities (ANOSIM).

OTUs representing < 0.005% of the population were removed and taxonomy was assigned using the RDP classifier. The relative abundance of each OTU was counted at different taxonomic levels. Then, bioinformatics analysis was mainly performed using QIIME (v1.7.0) and R packages (v3.2.0). The OTU table in QIIME was used to calculate OTU-level and β-diversity was assessed by principal coordinate analysis (PCoA). Cluster analysis and significant differences between samples were tested by ANOSIM.

### Statistical Analysis

Differences in the diarrhea incidence were tested by the χ^2^ contingency test. All other data were analyzed by ANOVA method using the GLM model in SAS 9.2 statistical software with repetition (cycle) as the statistical unit. A very significant difference between the means was defined as *p* ≤ 0.01 and significant difference between the means was defined at *p* ≤ 0.05, while a trend for the significance between the means was designated at 0.05 < *p* ≤ 0.10.

## Results

### Growth Performance and Diarrhea Rate

As shown in Table 2, pigs in the CE group had higher ADG during days 0–14 and days 0–28 and higher body weight at days 14 and 28 than pigs in the CON group (*p* < 0.05) without significant difference compared with the CTC. The CE diet did not significantly change the ADFI of pigs, but in the early stage and the whole period of the experiment, the F:G ratio and the diarrhea rate from days 0 to 14 were significantly reduced compared with the CON diet (*p* < 0.05). However, there was no differences in growth performance or diarrhea rate between CE and CTC.

**TABLE 2:** Effect of CE on growth performance and diarrhea rate of weaned piglets.

Items	CON	CE	CTC	SEM	*p*-value
Day 0 BW (kg)	7.25	7.24	7.25	0.04	0.97
Day 14 BW (kg)	10.97^b^	11.45^a^	11.34^a^	0.12	0.03
Day 28 BW (kg)	15.88^b^	16.80^a^	16.66^a^	0.21	0.01
**Days 0–14**					
ADG (g)	265.71^b^	300.98^a^	292.14^a^	7.54	0.01
ADFI (g)	441.43	456.09	460.01	9.33	0.20
F:G	1.66^a^	1.51^b^	1.57^b^	0.02	<0.01
Diarrhea rate (%)	3.46^a^	1.34^b^	1.23^b^	0.39	<0.01
**Days 15–28**					
ADG (g)	351.07	382.32	379.91	17.24	0.39
ADFI (g)	658.21	655.27	660.45	15.47	0.97
F:G	1.89	1.73	1.74	0.06	0.13
Diarrhea rate (%)	1.00	1.00	1.23	0.57	0.95
**Days 0–28**					
ADG (g)	308.39^b^	341.65^a^	336.03^a^	7.52	0.02
ADFI (g)	549.82	560.18	560.22	5.30	0.31
F:G	1.79^a^	1.65^b^	1.67^b^	0.03	0.01
Diarrhea rate (%)	2.23	1.17	1.23	0.39	0.13

*SEM, standard error of the mean (n = 8).*

*^a,b^Different superscripts within a row indicate a significant difference (p < 0.05).*

### Nutrient Digestibility

The effects of CE on nutrients in weaned piglets are shown in Table 3. Compared with the CON, the apparent total tract digestibility of ADF and NDF was significantly increased in pigs fed CE diet in the early and late stages of experiment (*p* < 0.05), and there was no significant difference between the CE and CTC groups.

**TABLE 3:** Effects of CE on total intestinal apparent digestibility of nutrients in weaned piglets (%).

Items	CON	CE	CTC	SEM	*p*-value
**Days 0–14**					
DM	81.51	81.16	82.01	0.40	0.34
OM	83.93	83.18	83.53	0.33	0.31
CP	76.14	75.12	76.87	0.55	0.12
GE	81.61	81.11	82.08	0.32	0.13
ADF	56.82^b^	65.20^a^	65.99^a^	1.75	<0.01
NDF	40.92^b^	54.74^a^	51.05^a^	2.44	<0.01
**Days 15–28**					
DM	81.17	80.36	80.58	0.27	0.13
OM	83.29	82.72	82.54	0.29	0.19
CP	75.19	76.31	76.22	0.57	0.33
GE	81.28	80.39	80.46	0.35	0.17
ADF	59.81^b^	64.38^a^	64.44^a^	1.25	0.03
NDF	46.90^b^	54.86^a^	55.87^a^	2.01	0.01

*SEM, standard error of the mean (n = 8).*

*^a,b^Different superscripts within a row indicate a significant difference (p < 0.05).*

### Immunity and Antioxidant Properties

As shown in Table 4 that compared with the CON, CE induced higher serum IgA concentrations in weaned piglets on day 14 (*p* < 0.05) and higher IgG on day 28, which was not significantly different from the CTC. Table 5 shows that pigs fed CE had higher serum SOD concentration on day 14 (*p* < 0.05) compared with the CON and was not significantly different from the CTC. The CE group showed increased serum GSH-Px concentration of pigs on day 28 compared with the CON (*p* < 0.05) without significant difference compared with the CTC group. Moreover, the CE diet tended to decrease the serum MDA concentration in weaned piglets compared with the CON diet (0.05 < *p* ≤ 0.10).

**TABLE 4:** Effects of dietary CE supplementation on serum immunological traits of weaned pigs.

Items	CON	CE	CTC	SEM	*p*-value
**Day 14**					
IgA (g/L)	1.00^b^	1.17^a^	1.14^a^	0.04	0.01
IgG (g/L)	6.93	7.36	7.99	0.33	0.11
IgM (g/L)	0.57	0.57	0.64	0.02	0.09
**Day 28**					
IgA (g/L)	2.81	2.59	2.73	0.08	0.23
IgG (g/L)	8.34^b^	9.73^a^	9.85^a^	0.40	0.03
IgM (g/L)	0.10	0.11	0.12	0.01	0.16

*SEM, standard error of the mean (n = 8).*

*^a,b^Different superscripts within a row indicate a significant difference (p < 0.05).*

**TABLE 5:** Effect of CE on antioxidant parameters of weaned piglets.

Items	CON	CE	CTC	SEM	*p*-value
**Day 14**					
GSH-Px (U/ml)	748.05	752.22	768.75	24.47	0.82
MDA (nmol/ml)	4.02	3.32	2.87	0.30	0.05
SOD (U/ml)	119.78^b^	133.13^a^	140.16^a^	4.03	<0.01
T-AOC (U/ml)	8.48	9.40	10.11	0.62	0.21
**Day 28**					
GSH-Px (U/ml)	297.68^b^	319.53^a^	320.37^a^	5.44	0.02
MDA (nmol/ml)	5.84	4.38	4.28	0.49	0.07
SOD (U/ml)	180.43	180.87	180.29	5.70	0.76
T-AOC (U/ml)	7.75	7.80	8.08	0.34	0.76

*SEM, standard error of the mean (n = 8).*

*^a,b^Different superscripts within a row indicate a significant difference (p < 0.05).*

### Volatile Fatty Acid Composition

VFA profiles of ileal, cecal, and colonic segments are shown in Table 6. Pigs fed CE diet had increased acetic acid, propionic acid, and total VFA contents in the cecum and colon compared with CON (*p* < 0.05) with no significant difference compared with the CTC.

**TABLE 6:** Effect of CE on VFA content in different intestinal segments of weaned piglets (mg/kg).

Items	CON	CE	CTC	SEM	*p*-value
**Ileum**					
Acetic acid	489.06	481.36	491.70	43.59	0.98
Propionic acid	129.42	136.85	144.32	16.67	0.82
Isobutyric acid	16.98	17.45	15.97	0.68	0.32
Butyric acid	62.86	68.16	69.00	5.70	0.72
Isovaleric acid	7.13	8.83	7.49	1.45	0.69
Valeric acid	8.09	8.24	8.91	0.80	0.74
Total VFA	713.51	720.89	737.45	44.56	0.93
**Caecum**					
Acetic acid	3,433.76^b^	4,514.55^a^	4,449.69^a^	309.32	0.04
Propionic acid	2,014.41^b^	2,792.03^a^	2,809.36^a^	223.22	0.04
Isobutyric acid	82.18	55.62	91.19	13.09	0.17
Butyric acid	1, 056.40	1, 194.49	1, 233.27	94.99	0.41
Isovaleric acid	90.29	70.91	90.73	9.92	0.30
Valeric acid	193.99	180.81	210.94	22.67	0.65
Total VFA	6,871.04^b^	8,808.40^a^	8,885.18^*a*^	564.34	0.04
**Colon**					
Acetic acid	2,538.82^b^	3,141.48^a^	3,305.58^a^	186.45	0.03
Propionic acid	1,616.77^b^	2,083.41^a^	2,207.99^a^	140.07	0.02
Isobutyric acid	90.24	120.99	113.61	12.31	0.22
Butyric acid	1, 142.52	1, 222.97	1, 295.80	118.56	0.67
Isovaleric acid	161.77	192.08	185.77	11.10	0.16
Valeric acid	306.73	327.35	300.58	22.84	0.69
Total VFA	5,856.84^b^	7,088.28^a^	7,409.33^a^	326.98	0.01

*SEM, standard error of the mean (n = 8).*

*^a,b^Different superscripts within a row indicate a significant difference (p < 0.05).*

*Total VFAs = Acetate + Propionate + Butyrate + Valerate + Isobutyrate + Isovalerate.*

### Intestinal Morphology

As shown in Table 7, pigs in CE group had lower crypt depth and higher villus height and villus height-to-crypt depth ratio in jejunum compared with that of CON (*p* < 0.01). The duodenum villus height of pigs in the CE group was significantly higher than that in the CON (*p* < 0.05) with no significant difference compared with the CTC.

**TABLE 7:** Effect of CE on intestinal morphology of weaned piglets.

Items	CON	CE	CTC	SEM	*p*-value
**Duodenum**					
Villus height (μm)	258.58^b^	302.41^a^	302.18^a^	12.16	0.03
Crypt depth (μm)	252.26	220.42	246.12	16.26	0.37
Villus height/Crypt depth	1.08	1.40	1.26	0.10	0.10
**Jejunum**					
Villus height (μm)	271.33^b^	356.53^a^	296.00^b^	11.52	<0.01
Crypt depth (μm)	200.69^a^	159.42^b^	183.48^a^	7.45	<0.01
Villus height/Crypt depth	1.37^b^	2.25^a^	1.62^b^	0.11	<0.01
**Ileum**					
Villus height (μm)	255.62	291.54	264.89	11.78	0.11
Crypt depth (μm)	151.08	141.07	150.93	4.97	0.29
Villus height/Crypt depth	1.70	2.07	1.76	0.11	0.06

*SEM, standard error of the mean (n = 8).*

*^a,b^Different superscripts within a row indicate a significant difference (p < 0.05).*

### Cecal Microbiota

**FIGURE 1:**
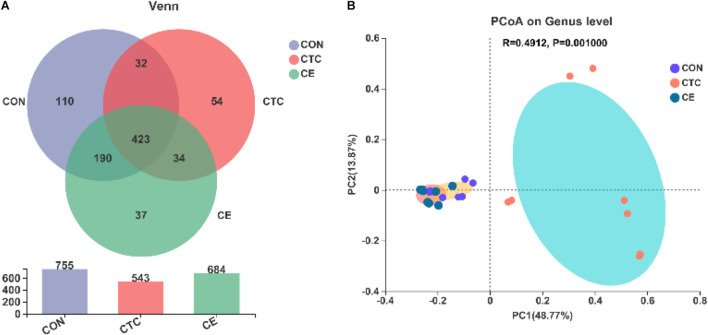
Effect of compund enzymes on cecal OTU of pigs **(A)**. β-diversity of cecal microbes in piglets **(B)**. The individual minipig was regarded as the experimental unit, *n* = 8 for CE, *n* = 8 for CON, and *n* = 8 for CTC. CE, compund enzyme group; CON, basal diet group; CTC, chlortetracycline group.

Venn diagrams showed that there were 684, 543, and 755 OTUs in cecal digesta samples of CE, CON, and CTC groups, respectively, of which 423 OTUs were shared and 201 OTUs were unique (Figure 1A). The β-diversity of bacterial community between CON, CTC, and CE groups was presented with PCoA (Figure 1B). The PCoA with the Bray–Curtis distance indicated that the samples of the CON group gathered together and clearly separated from the samples of the CE and CTC groups.

**FIGURE 2:**
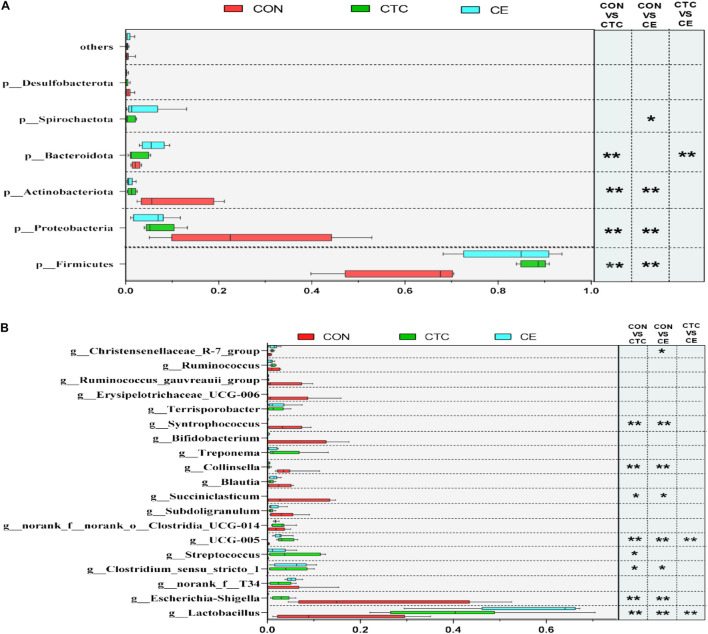
Different microbiota comparison by the Student’s *t*-test on phylum **(A)** and genus **(B)** of cecum. LEfSe analysis showed significantly changed bacteria between CON, CE, and CTC group. The individual minipig was regarded as the experimental unit (*n* = 8). CE, compund enzyme group; CON, basal diet group; CTC, chlortetracycline group. *p < 0.05 and **p < 0.01 represent significant difference, respectively.

At the phylum level (Figure 2A), the dominant phyla of cecum in three groups were *Firmicutes* and *Proteobacteria*. The relative abundance of *Firmicutes* in cecum was significantly increased in piglets fed CE and CTC diets compared with CON diet (*p* < 0.01). Feeding CE and CTC diet significantly decreased the relative abundance of *Proteobacteria* and *Actinobacteriota* in cecum (*p* < 0.01). On day 28, the top three genera in the CON group were *Escherichia-Shigella*, *Lactobacillus*, and *Succiniclasticum* (Figure 2B), while those in CTC group were *Lactobacillus*, *Clostridium_sensu_stricto_1*, and *Streptococcus*, and *Lactobacillus*, *norank_f_T34*, and *Clostridium_sensu_stricto_1* in CE group. At the genus level, the CE supplementation significantly decreased the relative abundance of *Escherichia_Shigella*, *Collinsella*, and *Syntrophococcus* in cecum compared with CON (*p* < 0.01). Piglets fed CE diet had greater relative abundance of *Lactobacillus* than those fed the other two diets (*p* < 0.001). In addition, supplementation of CE and CTC diet reduced (*p* < 0.05) the relative abundance of *Succiniclasticum* in cecum when compared with the CON diet.

**FIGURE 3:**
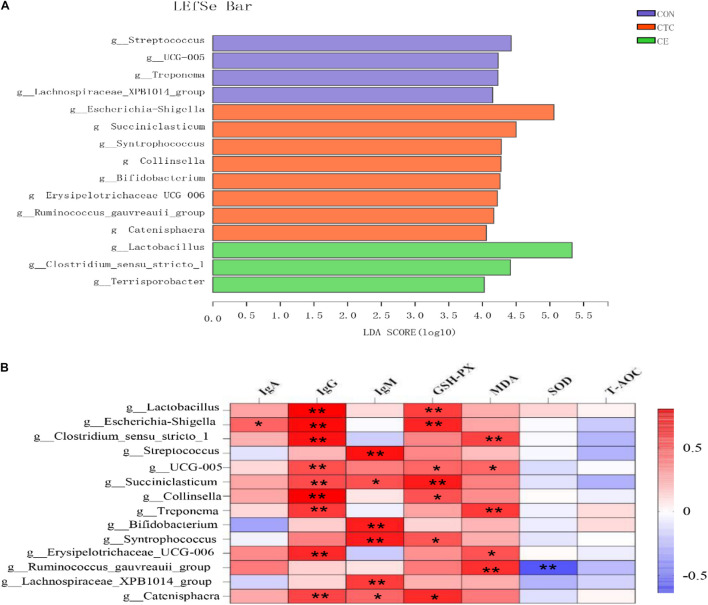
Identification of the most differentially abundant genera in cecum **(A)**. The plot is generated from Linear Discriminant Analysis Effect Size (LEfSe) analysis with CSS-normalized OTU table and displays taxa with LDA scores above 4 and *p*-values below 0.05. Genera enriched in the samples with CTC diet are indicated with red bars, genera enriched in the samples with CON diet are indicated with blue bars, and genera enriched in the samples with CE diet are indicated with green bars. The individual minipig was regarded as the experimental unit (*n* = 8). CE, compund enzyme group; CON, basal diet group; CTC, chlortetracycline group. Correlation analysis between serum biochemical parameters and cecal microorganisms **(B)**. *p < 0.05 and **p < 0.01 represent significant difference, respectively.

The LEfSe analysis was used to identify the significantly different bacteria at the genus level between the three treatments (Figure 3A). A total of 15 cecal genera were identified to be significantly different between the three groups, namely, 3 genera from CE, 8 genera from CON, and 4 genera from CTC. The relative abundance of *g_Lactobacillus, g_Clostridium_sensu_stricto*, and *g_Terrisporobacter* in cecum was increased in piglets fed with the CE diet; the relative abundance of *g_Escherichia-Shigella, g_Succiniclasticum, g_Collinsella, g_Syntrophococcus, g_Bifidobacterium, g_Erysipelotrichaceae_UCG006, g_Ruminococcus_gauvreauii_group*, and *g_Catenisphaera* was increased in piglets fed with the CON diet, whereas the relative abundance of *g_Streptococcus*, *g_Treponema, g_UCG-005*, and *g_Lachnospiraceae_XPB1014_group* was increased in cecum of piglets fed with the CON diet.

Correlation analysis between serum biochemical parameters and cecal microorganisms has been shown in Figure 3B, wherein the bacteria including *g_Lactobacillus, g_Escherichia-Shigella, g_Clostridium_sensu_stricto_1, g_Streptococcus, g_ UCG-005, g_Succiniclasticum, g_Collinsella, g_Treponema, g_Bifidobacterium, g_Syntrophococcus, g_Erysipelotrichaceae_UCG006, g_Ruminococcus_gauvreauii_ group, g__Lachnospiraceae_XPB1014_group*, and *g_Catenisphaeras* had a strong positive correlation with the concentration of IgG (*p* < 0.01). In addition, the bacteria including *g_Lactobacillus* and *g_Escherichia_—_Shigellag_Succiniclasticum* had a strong positive correlation with the GSH-Px (*p* < 0.01); the bacteria including g_Clostridium_sensu_stricto_1, g_Treponema, and g_Ruminococcus_gauvreauii_group had a strong positive correlation with the MDA (*p* < 0.01).

## Discussion

Adding multi-enzyme in the corn–soybean meal diet not only increased the ADG and G:F, but also increased the ATTD of ADF and NDF in the early and late stages of the experiment. However, dietary treatments had no significant effect on the ATTD of GE and the reason could be that the NSP enzyme acting as fiber-degrading enzyme has greater effects on fiber digestibility in ileum but no other nutrients such as fat and protein digestibility; therefore, no change in ATTD of GE was detected (Li et al., 2018). However, NSP can release more nutrients from the cell wall of grain, while neutral protease can decompose soybean antigen proteins in soybean meal, which are difficult for mammals to decompose into small peptides and amino acids (Meinlschmidt et al., 2016; Munyaka et al., 2016). Since the small peptides and amino acids are easily absorbed, the supplementation of compund enzyme consequently induced improved utilization efficiency of feed and promoted growth of piglets. Our study is consistent with previous studies in that CE promoted the growth of piglets by increasing the conversion rate of nutrients in the feed (Zhang et al., 2014; Li et al., 2018). Tsai et al. (2017) reported that adding both xylanase and β-glucanase into nursery diets with 30% corn DDGS improved ADG from days 7 to 35. However, according to the meta-analysis of Torres-Pitarch et al. (2017), 39% of studies showed that CE had no effects on pig growth performance, while 61% of studies showed that CE could improve pig growth performance. There are many factors affecting the growth performance of pigs, such as the proportion of CE, active ingredients, ages and breeds of pigs, feeding environment, and so on. In the future, it will be important to accurately predict the type and concentration of enzymes added to the diet according to different diets and growth stages of pigs.

Immunoglobulins are abundant in serum, have antibody activity or chemical structure similar to antibody molecules, and consist of two identical light and heavy chains linked by disulfide interchain bonds (Schroeder and Cavacini, 2010). Immunoglobulins play an important role in participating in antigen response and improving anti-immune function of weaned piglets. Long et al. (2021) found that CE increased serum immunoglobulins (IgA, IgG, and IgM) of pigs, indicating that CE could enhance the immune function of weaned pigs. He et al. (2020) reported that the CE could enhance the sIgA content in jejunal mucosa, which indicated that CE could help improve intestinal immune function of weaned pigs. Our study is also consistent with the results of previous studies in that CE increased serum IgA and IgG levels of piglets, reflecting that CE can improve the disease resistance of piglets. The CE could probably decompose the NSP in the soybean diet into oligosaccharides, which could help improve the immune function in pigs (Agyekum et al., 2015). The normal growth of animals can be guaranteed by maintaining the relative stability between systematic oxidation and antioxidant status. As the main product of lipid peroxidation, MDA will induce oxidative stress response when it was excessively produced and deposited (Ozdemir et al., 2007). SOD, GSH-Px, and other antioxidant enzymes can use the chain reaction mechanism to reduce the free radical reaction and protect animal from the damage of pro-oxidants (Benzie, 2003; Minelli et al., 2009; Yin et al., 2013). In the present study, dietary CE supplementation showed increased concentrations of antioxidant enzymes including SOD and GSH-Px in serum and decreased serum MDA concentration, which indicated that CE may play an important role in eliminating reactive free radicals and alleviating oxidative stress response. This finding was partly consistent with the study of Long et al. (2021) who reported that dietary CE supplementation showed increased serum SOD and CAT content, and reduced serum MDA content. Additionally, Han et al. (2017) found that adding CE containing NSP enzyme and acid protease to piglet diet with antibiotics could significantly increase serum GSH-Px activity and significantly reduce MDA levels. Obviously, the improved antioxidant capacity may lead to reduced damage in intestinal barrier and alleviation of diarrhea (Huang et al., 2015). The positive effects of CE could be explained by reduced anti-nutritional factors in soybean meals due to the neutral protease addition, which may promote the healthy development of the intestinal tract, increase the colonization of beneficial bacteria in the intestinal tract, and reduce the post-intestinal fermentation, thereby improving the antioxidant capacity of piglets.

Microbial fermentation mainly occurs in the cecum and colon of pigs and play an important role in the intestinal health of pigs. It can produce a variety of VFAs, mainly including formic acid, acetic acid, propionic acid, and butyric acid, which can effectively inhibit the reproduction of harmful bacteria and enhance the absorption of intestinal nutrients (Högberg and Lindberg, 2006). In our study, the acetic acid, propionic acid, and total VFA concentrations in cecum and colon were significantly increased in pigs fed the diet supplemented with compound enzyme than those without enzymes. Similarly, Long et al. (2021) reported that dietary CE supplementation tended to increase acetic acid content in colon. Yi et al. (2013) also found that dietary CE supplementation could effectively enhance the VFA contents and improve the health status in weaned pigs. According to the previous studies, acetic acid and propionic acid contents in the large intestine of piglets increased due to dietary CE supplementation; one explanation could be the improved microflora profiles since acetic acid and propionic acid are the main metabolites of microflora in the large intestine (Yi et al., 2013; Zhang et al., 2014). Since VFA has been linked with intestinal health of animals, a higher VFA concentration also explained the improvement in growth performance of piglets.

The integrity of intestinal morphology and structure is a necessary condition to maintain the growth and healthy status of piglets. The ratio of intestinal villus height to crypt depth can directly reflect nutrient digestibility and gastrointestinal absorption function. A higher villus height-to-crypt depth ratio was more favorable for pigs to digest and absorb nutrient (Lm et al., 2003). However, weaning stress can significantly reduce intestinal villus height and villus atrophy can lead to intestinal cell death and reduce cell renewal rate (Wang et al., 2011). In our study, supplementation of CE increased the villus height of duodenum and jejunum as well as the villus height-to-crypt depth ratio in ileum. This finding was in agreement with the study of Long et al. (2021), who reported that CE could increase the villus height and the villus height-to-crypt depth ratio in ileum of weaned pigs. Jiang et al. (2015) also found that CE could improve ileal histology and intestinal health of weaning piglets. The reason for the current finding might be that CE can improve nutrient digestion and absorption in the small intestine and improve the intestinal integrity due to the increased nutrient supply to the intestinal tract (Wijtten et al., 2011).

This study also found that CE increased the relative abundance of *Firmicutes* and *Lactobacillus* in cecum, while the relative abundance of *Proteobacteria* and *Actinobacteriota* and *Escherichia-Shigella* were reduced by CE. These results indicated that CE had a good regulatory effect on the intestinal microflora of piglets. Moreover, an increase in the relative abundance of lactic acid bacteria or a decrease in the relative abundance of *Escherichia-Shigella* may be more beneficial for the intestinal health of weaned piglets and contributed to an improved growth performance in the CE group. Similar to our results, Long et al. (2021) found that dietary CE supplementation increased the relative abundance of *Firmicutes*, *Bacilli*, and *Lactobacillus* in cecum and colon. The changed microbial profiles induced by dietary treatments could be that the combination of protease, amylase, and cellulase reduced the posterior intestinal fermentation of piglets, inhibited the reproduction of harmful bacteria, and increased the abundance of beneficial bacteria. At the same time, the combination of exogenous amylase or β-glucanase with other enzymes can also reduce the relative abundance of harmful bacteria such as *Escherichia coli* in the intestinal tract of weaned piglets, which helps to reduce the diarrhea rate (Zhang et al., 2014; Jiang et al., 2015). Overall, these findings revealed that CE could be used to replace CTC to achieve a growth-promoting effect in some extent and may be even better than antibiotics in improving intestinal microflora in weaned piglets.

Members of *Firmicutes* can produce short-chain fatty acids by degrading carbohydrate, which are closely related to energy acquisition and immune response regulation in the body (Atarashi et al., 2011; Zhang et al., 2015). *Clostridium* has been reported to affect the accumulation of CD8^+^ IELs (intraepithelial lymphocytes) in the colon (Umesaki et al., 1999). Based on these studies, it is not hard to find that microbes can modulate various aspects of the immune system function. In our study, the correlation analysis between serum biochemical indexes and cecal microorganisms showed that *g_Lactobacillus*, *g_UCG-005*, *g_Succiniclasticum*, *g_Collinsella*, and *g__Catenisphaeras* had a positive correlation with the concentration of IgG and GSH-Px, while *g_Ruminococcus-gauvreauii-group* had a negative correlation with the concentration of MDA and SOD. Some families and genera of *Clostridiales* cannot only degrade oligosaccharides into butyrate, but also regulate the balance of intestinal microflora (Louis and Flint, 2009) and are mediated by G-protein-coupled receptors in immune cells. Some members of the *Lactobacillales*, *Clostridiales*, and *Bifidobacteriales* are metabolically capable of producing conjugated linoleic acid (Devillard et al., 2007), which has an ability to enhance animal immunity by increasing the activity of immunoglobulin in serum and antioxidant enzymes in liver of animals (Ramírez-Santana et al., 2011; Huang et al., 2017). Besides lactic acid and acetic acid production (Mitsuoka, 1990), *Bifidobacterium* can promote the production of cytokines, specific antibodies, and non-specific antibodies, which induced activated immunity and enhanced disease resistance (Moya-Pérez et al., 2017). Its mechanism of action may be that *Bifidobacterium* forms a defensive barrier through competitive inhibition, and its metabolites can increase the content of free cholic acid and inhibit the growth of pathogenic microorganisms by activating the body to produce catalase (Zuo et al., 2013). The study of Liu et al. (2017) showed that *Lactoales* and *Enterococcus faecalis* could increase the intestinal immune function of piglets and reduce the incidence of diarrhea and mortality of piglets. Similarly, studies have shown that daily probiotic strains containing *Bifidobacterium* and *Lactobacillus* treatment in mice can alter the inflammatory state of mice (Giacinto et al., 2005; Lyons et al., 2010; Karimi et al., 2012).

## Conclusion

In summary, supplementation of CE in diets effectively improved nutrient digestibility and immunity function in weaned piglets and increased relative abundance of *Lactobacillus* species in the cecum, which could contribute to the improved intestinal health, and consequently was associated with improved growth performance and reduced diarrhea rate of pigs. Overall, CE had comparative effects on growth performance, immunity, antioxidant capacity, and intestinal health of weaned piglets.

## Data Availability Statement

The datasets presented in this study can be found in online repositories. The names of the repository/repositories and accession number(s) can be found below: NCBI SRA database and Bioproject accession number: PRJNA760807 (https://www.ncbi.nlm.nih.gov/bioproject/PRJNA760807).

## Ethics Statement

The animal study was reviewed and approved by the Committee of Animal Care at Hunan Agricultural University (Changsha, China) (Permit Number: CACAHU 2021-01106).

## Author Contributions

ZL and XM: conceptualization, methodology, and software. LT, NL, XL, and FZ: literature collection. ZL and QJ: writing—original draft preparation. JC: writing—reviewing and editing. XM: funding acquisition. All authors contributed to the article and approved the submitted version.

## Conflict of Interest

The authors declare that the research was conducted in the absence of any commercial or financial relationships that could be construed as a potential conflict of interest.

## Publisher’s Note

All claims expressed in this article are solely those of the authors and do not necessarily represent those of their affiliated organizations, or those of the publisher, the editors and the reviewers. Any product that may be evaluated in this article, or claim that may be made by its manufacturer, is not guaranteed or endorsed by the publisher.

## Abbreviations

BW: body weight
ADG: average daily gain
ADFI: average daily feed intake
G:F: gain-to-feed ratio
DM: dry matter
OM: organic matter
CP: crude protein
GE: gross energy
ADF: acid detergent fiber
NDF: neutral detergent fiber
IgA: immunoglobulin A
IgG: immunoglobulin G
IgM: immunoglobulin M
SOD: superoxide dismutase
GSH-Px: glutathione peroxidase
T-AOC: total antioxidant capacity
MDA: malondialdehyde
VFAs: volatile fatty acids
SCFAs: short-chain fatty acids
BCFAs: branched-chain fatty acids
PCR: polymerase chain reaction
OTUs: operational taxonomic units
PCoA: principal coordinate analysis
LDA: linear discriminant analysis
LEfSe: linear discriminant analysis effect size.
